# Supplementary material for: Extract from the Macroalgae Ulva rigida Induces Table Grapes Resistance to Botrytis cinerea
Source: Foods. 2022 Feb 28;11(5):723. doi: 10.3390/foods11050723 (PMC8909532; doi:10.3390/foods11050723)
Supplement: Supplementary file 1 [file foods-11-00723-s001.zip › foods-1582913-supplementary.pdf]

Article

Extract from the macroalgae Ulva rigida induces table grapes resistance to *Botrytis cinerea*

Alon Shomron[1,2], Danielle Duanis-Assaf[2,3], Ortal Galsurker[2], Alexander Golberg[1*], Noam Alkan[2*]

[1] Porter School of Environment and Earth Sciences, Faculty of Exact Sciences, Tel Aviv University, Tel Aviv, Israel;
[2] Department of Postharvest Science of Fresh Produce, Agricultural Research Organization (ARO), Volcani Center, Rishon LeZion 7505101, Israel;
[3] Robert H. Smith Faculty of Agriculture, Food and Environment, the Hebrew University of Jerusalem, Rehovot, 76100, Israel;

1. FT-IR data analysis

The identification of the thermochemical hydrolysis extract from Ulva sp. was compared to the extract's features and values of previous studies and was calculated in the following manner:

the absolute difference between the mean of published data and scanned data ($|\Delta V|$) was divided by the mean of published data ($\frac{1}{n}\Sigma V_i$), and expressed as a percentage:

$$\%\ \text{similarity} = \left(1 - \frac{|\Delta V|}{\frac{1}{n}\Sigma V_i}\right) \times 100 \qquad (1)$$

Where:

N = Number of samples

$|\Delta V|$ = Absolute difference of FT-IR value and mean of previously published values

$\Sigma V_i/\text{n}$ = Mean of previously published values

Table S1. Comparisons of peak wavenumber (Ṽ) values gathered from the FT-IR scan compared with peaks gathered from previously published data of ulvan-extracts.

FT-IR scan peaks (Ṽ in cm^{-1})	previously published peaks (Ṽ in cm^{-1})	mean of published peaks (Ṽ in cm^{-1})	Similarity in peak location (%)	Assignment of area on the spectrum
3395	3446[a]<br>3340[b]<br>3420[e]	3402	99.86	O—H<br>Stretch
2937	2938[c]<br>2920[e]	2929	98.75	C—H<br>Stretch
1620	1646[d]<br>1599[c]<br>1644[e]	1629.6	99.6	C=O<br>v_{as}
1411	1420[b]<br>1423[c]<br>1444[e]	1429	99.15	C=O<br>v_{s}
1302	1322[b]	-	98.98	CH-OH<br>δ
1236	1260[e]<br>1256[a]<br>1215[c]	1243.6	99.5	S=O<br>v_{as}
1109	1058[e]<br>1052[a]<br>1055[d]	1055	96.46	C—O—C<br>Stretch
844	847[a]<br>845[b]<br>848[e]	846.6	99.79	C—O—S<br>Bonds<br>in axial position
784	785[b]	789.6	99.52	C—O—S<br>Bonds<br>in equatorial position

δ = bending, v = stretching, vas = asymmetric stretching

***a** Z. Zhang, F. Wang, X. Wang, X. Liu, Y. Hou, and Q. Zhang, "Extraction of the polysaccharides from five algae and their potential antioxidant activity in vitro," *Carbohydr. Polym.*, 2010, doi: 10.1016/j.carbpol.2010.04.031.

***b** G. Toskas, R. D. Hund, E. Laourine, C. Cherif, V. Smyrniotopoulos, and V. Roussis, "Nanofibers based on polysaccharides from the green seaweed Ulva Rigida," *Carbohydr. Polym.*, 2011, doi: 10.1016/j.carbpol.2010.12.075.

***c** T. T. T. Thanh, T. M. T. Quach, T. N. Nguyen, D. Vu Luong, M. L. Bui, and T. T. Van Tran, "Structure and cytotoxic activity of ulvan extracted from green seaweed Ulva lactuca," *Int. J. Biol. Macromol.*, 2016, doi: 10.1016/j.ijbiomac.2016.09.040.

***d** E. Hernández-Garibay, J. A. Zertuche-González, and I. Pacheco-Ruíz, "Isolation and chemical characterization of algal polysaccharides from the green seaweed Ulva clathrata (Roth) C. Agardh," *J. Appl. Phycol.*, 2011, doi: 10.1007/s10811-010-9629-0.

***e** H. Yaich *et al.*, "Effect of extraction procedures on structural, thermal and antioxidant properties of

ulvan from Ulva lactuca collected in Monastir coast," *Int. J. Biol. Macromol.*, 2017, doi: 10.1016/j.ijbiomac.2017.07.141.

Table S2. Changes in pH levels, sugar levels (Brix; TSS), titratable acidity

Grapes, *Vitis vinifera* cv. 'Scarlotta' were treated with water (control) or 1,000mg/L ulvan extract. pH levels, TSS, and acidity were tested before treatment and 2 and 4 days post-treatment. The results display as average and SE of three biological repeats, each performed in triplicates.

Properties / Treatment	pH	Brix (total soluble solids)	% Tartaric acid
Before treatment (Day 0)	4.51±0.06	18.73±0.40	0.22±0.01
1,000mg/L ulvan (Day 2)	4.67±0.08	20.00±0.09	0.23±0.02
1,000mg/L ulvan (Day 4)	4.59±0.09	19.47±0.27	0.27±0.02
DDW (control) (Day 2)	4.58±0.03	20.33±0.05	0.24±0.01
DDW (control) (Day 4)	4.73±0.09	19.37±0.24	0.21±0.02

The data display as the average of three repeats.

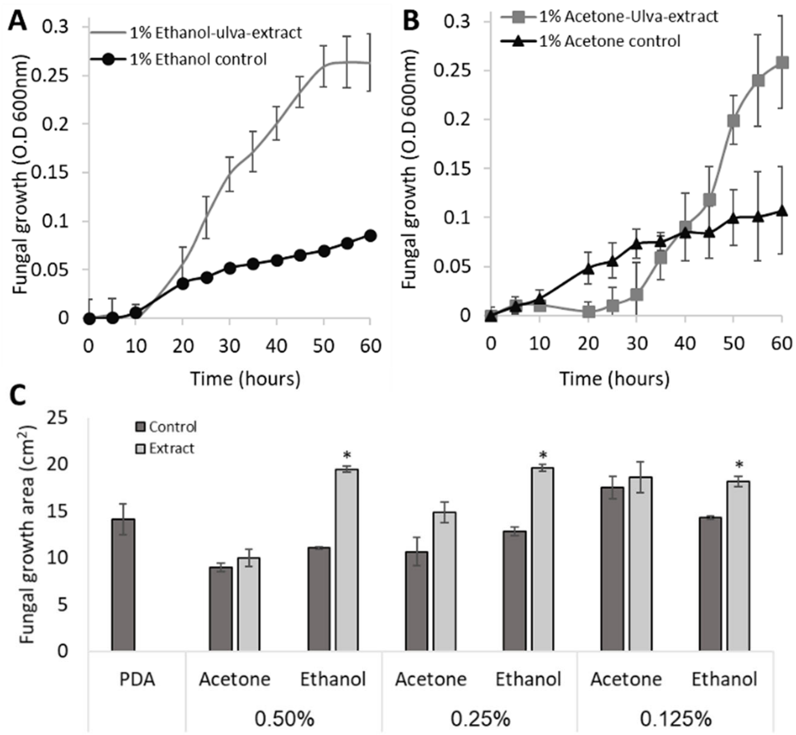

Figure S1. Ulva extracts promote *in vitro* growth of *B. cinerea***.** A-B. *B. cinerea* conidia were grown in the presence of SMB growth medium with or without *Ulva rigida* ethanolic (A) and acetonic (B) algal extracts at the concentration of 1% v/v. Optical density at 600nm was monitored across several days. Average and SE are presented. C. Fungal colony area after seven days growth on agar plates containing acetonic or ethanolic algal-extracts and their controls in various concentrations. Average and SE are presented. Asterisks indicate t-test significant difference between treatment and control ($p \leq 0.05$).

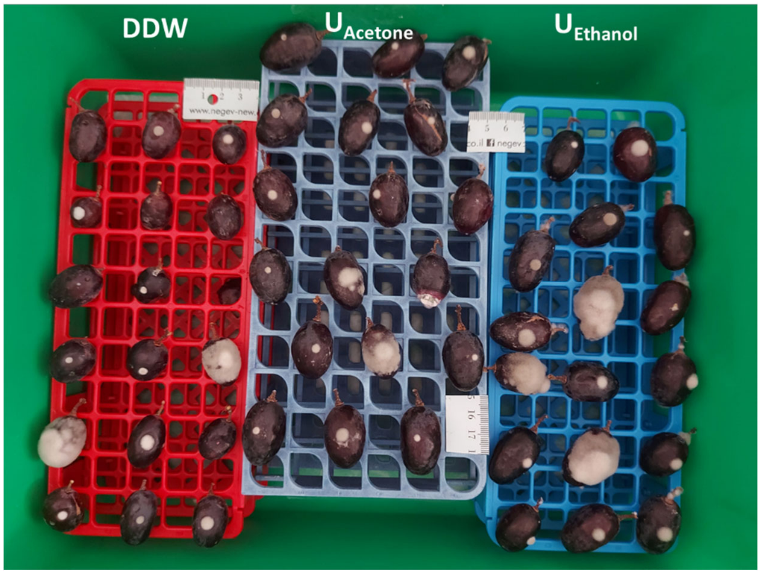

Figure S2. Grey mold development on grapes treated with ethanolic or acetonic extracts. Grapes were treated with either ethanolic or acetonic extracts of *Ulva rigida*, or double deionized water (DDW, control). Subsequently, the grapes were drop inoculated with $10^5 \cdot mL^{-1}$ *B. cinerea* conidia 2 days post algal treatment.

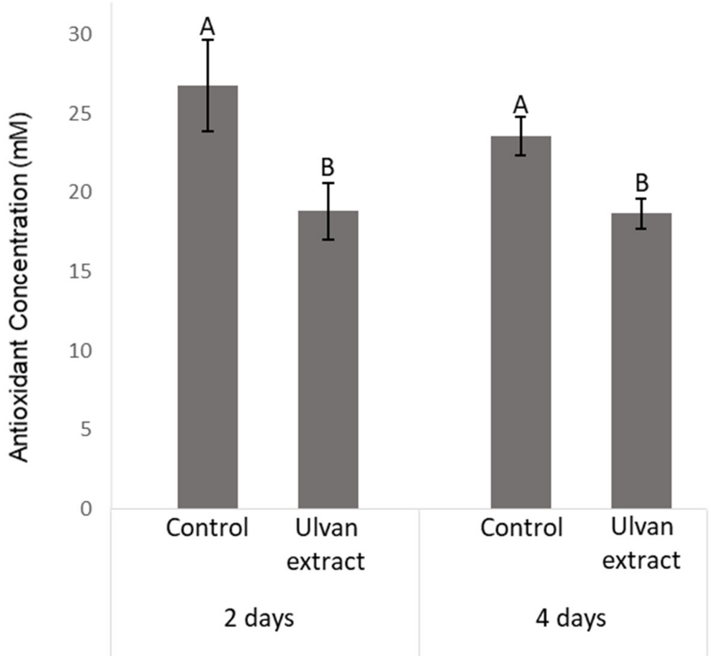

Figure S3. Total antioxidants in table grapes in response to ulvan treatment. Grapes, *Vitis vinifera* cv. 'Scarlotta' were treated with water (control) or 1,000mg/L ulvan extract. Peel samples were taken 2 and 4 days post-treatment for DPPH analysis. The results display as average and SE of three biological repeats, each performed in triplicates. Different letters indicate significant difference between treatments (*p* ≤ *0.05)*.
